# The impact of Vitreo-Macular interface abnormalities on the response to Anti-VEGF therapy for centre involving diabetic macular oedema

**DOI:** 10.1007/s00417-024-06518-6

**Published:** 2024-05-21

**Authors:** Matthew Maguire, Dah Laidlaw, Nigel Davies, Christopher Hammond

**Affiliations:** 1 Department Academic Ophthalmology, Guy’s and saint Thomas’ NHS Trust, London, SE1 7EH UK; 2 Department Ophthalmology, Guy’s and saint Thomas’ NHS Trust, London, UK

**Keywords:** Diabetic Macular Oedema, DMO, DME, OCT; ocular coherence tomography, vitreomacular interface abnormality; VMIA, ERM; epiretinal membrane, VMT; vitreomacular traction, Anti-VEGF

## Abstract

**Background:**

The influence of Vitreomacular Interface Abnormalities (VMIA) such as Epiretinal Membrane (ERM) and/or vitreomacular traction (VMT) on the response of patients with Centre Involving Diabetic Macular Edema (CIDME) to standard of care Anti-VEGF medications is under-researched.

The aims of this study were:

1. To determine the incidence of VMIA at baseline and 12 months amongst treatment naive patients commencing anti-VEGF treatment
2. To compare the response to Anti-VEGF medications at 3 monthly intervals for 12 months in a large cohort of patients with and without VMIA on their baseline OCT scan.

Response was determined in terms of: number of injections, central macular thickness and visual acuity.

**Methods:**

A retrospective case notes review of treatment naïve patients with newly diagnosed CIDME. Included patients had been commenced on intravitreal Anti-VEGF injections (ranibizumab or aflibercept) at a single centre.

Inclusion criteria were: treatment naïve DME patients with a CMT of 400μ or more receiving anti-VEGF treatment with at least 12 months follow up and in whom macular OCT scans and visual acuity (VA) measurements were available within two weeks of baseline, 3, 6, 9 and 12 months.

Exclusion criteria included: previous intravitreal therapy, previous vitrectomy, cataract surgery during the follow-up period, concurrent eye conditions affecting vision or CMT.

**Results:**

119 eyes met the inclusion criteria and underwent analysis. Groups were comparable in their baseline demographics. Baseline CMT measurements were comparable at baseline (417μ and 430μ in the No-VMIA and VMIA groups respectively) and improved to approximately 300μ in both groups. From 6 months CMT continued to improve in the no-VMIA while progressively deteriorating in the VMIA group. Change in CMT was statistically different at 12 months between the 2 groups (108μ and 79μ, *p*= 0.04). There was a mean of 7 injections after 12 months.

**Conclusion:**

Our study has shown a 46% incidence of VMIA amongst patients newly diagnosed with centre involving DME undergoing treatment with anti-VEGF injections. We have also demonstrated a significant difference in CMT and VA response to anti-VEGF treatment in patients with and without VMIA. Initial response was similar between the 2 groups up until 6 months. From 6 to 12 months significant differences in treatment response emerged.

Differences in clinical response between patients with and without VMIA may help guide further prospective controlled studies and optimise treatment strategies.

**Supplementary Information:**

The online version contains supplementary material available at 10.1007/s00417-024-06518-6.


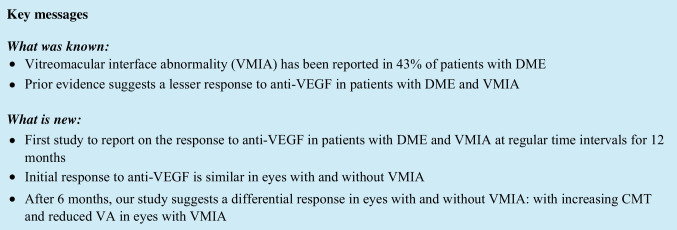


## Introduction

VMIA has been reported to be present on SD-OCT in 43% of patients with CIDME [1]. The influence of Vitreomacular Interface Abnormalities (VMIA) such as Epiretinal Membrane (ERM) and/or vitreomacular traction (VMT) on the response of patients with Centre Involving Diabetic Macular Edema (CIDME) to standard of care Anti-VEGF medications is under-researched; the 3 published series on this subject have however suggested that there is a lesser response to anti-VEGF in patients with DME and VMIA compared to those with no features suggestive of traction.

These studies included 24, 11 and 77 patients and only report change from baseline at single end-points of 1, 3 and 12-months respectively [1–3].

Therefore, while these outcomes have reported the deleterious effect of VMIA on visual acuity (VA) and central macular thickness (CMT) in eyes with DME treated with anti-VEGF, there is an absence of large studies charting the chronological response over regular time periods [1] with extended follow up periods.

It has also been suggested that vitrectomy may be an appropriate therapeutic option in patients with VMIA, but these data pre-date the anti-VEGF era [4]. A better understanding of disease response may provide greater insight into treatment strategies to optimise patient outcomes.

Anti-VEGF agents are the current standard of care treatment for patients with CIDME [5]. Ranibizumab and aflibercept have been shown to be effective treatment options [6] with an average of 7-9 injections required in the first year.

The aims of this single centre retrospective chart and scan review study were:

1. To determine the incidence of VMIA at baseline and 12 months amongst treatment naive patients commencing anti-VEGF treatment for CIDME at one centre
2. To compare the response to anti-VEGF medications at 3 monthly intervals for 12 months in a large cohort of patients with and without VMIA on their baseline OCT scan. Response was determined in terms of: number of injections, central macular thickness and visual acuity.

## Methods

This was a retrospective case notes review of treatment naïve patients with newly diagnosed CIDME. Included patients had been commenced on intravitreal Anti-VEGF injections at a single centre, St. Thomas’ Eye Hospital Unit, London, between January 2014 and December 2018.

Inclusion criteria were: treatment naïve DME patients with a CMT of 400μm or more receiving anti-VEGF treatment with at least 12 months follow up and in whom macular OCT scans and visual acuity (VA) measurements were available within two weeks of baseline, 3, 6, 9 and 12 months.

Exclusion criteria included: previous intravitreal therapy, previous vitrectomy, cataract surgery during the follow-up period, concurrent eye conditions affecting vision or CMT such as: BRVO; CRVO; AMD; active uveitis; amblyopia or advanced glaucoma. Prior focal macular laser was not an exclusion criterion.

Only a single eye was included from any one patient, in cases where both eyes were potentially eligible the eye treated first was included.

Patients treated with ranibizumab received doses at monthly intervals followed by a treat and extend pathway, extending the inter treatment interval by 1-month at a time if vision was stable within 5 letters and there was no evidence of CMT increase on OCT.

Patients treated with aflibercept were scheduled for 5 injections at monthly intervals followed by treatment at 8 week intervals for the rest of the first year of treatment.

Baseline demographics were collected at the time of first injection including: age, laterality, type of diabetes, diabetes duration, grade of retinopathy, VA, CMT and presence or absence of VMIA.

At 3, 6 and 9-month appointments VA and CMT measurements were recorded.

At the 12-month appointment, VA, CMT, total injection number and presence or absence of VMIA were recorded.

Retinopathy was graded as background, moderate, active proliferative or stable proliferative [7].

VA was recorded in, or transformed to, number of Early Treatment of Diabetic Retinopathy Score (ETDRS) letters for statistical analysis. Snellen VA was converted to logMAR by calculating the logarithm of the decimal of the inverted Snellen fraction. LogMAR scores were converted to number of ETDRS letters by the formula 85 – (logMAR/0.02).

OCT scans were performed using Heidelberg Spectralis SDOCT (Heidelberg engineering, Heidelberg, Germany), employing a 30x30 grid protocol with 60 micrometre line spacing.

VMIA was defined on OCT scan images as the presence of epiretinal membrane (ERM) or Vitreomacular traction (VMT) in the macular area, demarcated by the ETDRS grid overlay when centred over the fovea (see image 1).

ERM was defined as the presence of a hyperreflective band anterior to the ILM layer with underlying corrugation of the inner retinal layers [8].

VMT was defined as partial cortical vitreous detachment with persistent foveal attachment and associated foveal anatomical distortion.

Vitreomacular Adhesion (VMA) was defined as elevated cortical vitreous above the retinal surface with attachment at the fovea without distortion of foveal anatomy [9]. VMA was not classified as VMIA in this study.

Based on OCT scans at baseline and 12 months the presence or absence of VMIA was recorded.

OCT scans were reviewed by a single examiner (MM) and, in ambiguous cases, referred to a senior clinician (AL) for final classification.

CMT was recorded as the average thickness of the central 1mm subfield of the ETDRS grid when centred on the fovea. The Heidelberg auto-rescan algorithm was used to ensure positional matching of baseline, 3, 6, 9 and 12-month scans. All scans were manually checked to ensure accurate foveal centration and correct demarcation of retinal layers, with manual correction as necessary.

The study was considered a service evaluation by the audit review board of Guy’s and St Thomas’ NHS Foundation Trust therefore, research and ethics committee approval were not required.

Results were analysed on Stata Statistical Software: Release 16.0 for Mac, Sep 2019 (College Station, TX: Statacorp LLC).

Descriptive statistics were used to calculate demographic data, presented as an entire cohort and subdivided by the presence or absence of VMIA. Change in CMT was calculated at 3 monthly intervals from baseline. Means with standard deviation (SD) and Medians with inter quartile range (IQR) were presented as appropriate to the data distribution.

Baseline demographics were compared using the 2-sample t-test for groups and Mann-Whitney U-test for parametric and non-parametric continuous data. Categorical data were compared using Chi squared and Fisher’s exact test as appropriate to group sizes. Ordinal data were analysed using the Wilcoxon rank-sum test.

*P*-values ≤0.05 were taken as statistically significant.

## Results

264 patients were identified from injection records. 47 cases were lacking OCT scan data and 73 cases were missing sufficient data in the patient notes. 15 cases were recorded incorrectly as DME (9 cases of retinal vein occlusion and 5 cases of neovascular age related macular degeneration). 5 patients had previously undergone vitrectomy and 3 patients underwent cataract surgery during the follow-up period. 3 patients had received previous treatment with anti-VEGF injections.

In total from 264 cases, 146 were excluded and 119 met the inclusion criteria and were included in further analysis.

For the 119 study eyes, the demographic results and baseline differences between groups with and without VMIA are presented in Table 1.

**Table 1:** Baseline demographics for the entire cohort and presented by presence or absence of VMIA. (R1 = background, R2 = moderate, R3s = stable proliferative, R3a = active proliferative, PDR = combined proliferative)

	All subjects	No VMIA	VMIA	P- value
Number of subjects (%)	119	65 (54%)	54 (46%)	
Mean age in years (SD)	62 (11)	61 (9.5)	63 (12)	0.35
Median duration of Diabetes in years (IQR)	15 (9-21)	19 (10-21)	13 (5-21)	0.18
Laterality – Right eye (%)	68 (57%)	39 (60%)	29 (54%)	0.49
Number of Type 2 DM patients (%)	114 (96%)	64 (98%)	50 (93%)	0.11
Aflibercept treated (n)	88% (105)	88% (57)	89% (48)	0.84
12 month mean Injection number (SD)	7 (2.3)	7 (2.3)	7 (1.9)	0.75
Retinopathy grade R1, R2, R3s, R3a (n) [PDR]	38, 51, 25, 4 [29]	26, 28, 9, 1 [10]	12, 23, 16, 3 [19]	**0.05**
(%) R2, R2, R3s, R3a [PDR]	32%, 43%, 21%, 3% [24%]	41%, 44%, 14%, 2% [16%]	22%, 43%, 30%, 6% [36%]	

*SD* standard deviation, *IQR* Interquartile range

In summary, the groups were comparable in their baseline demographics. There was a statistical difference in the grade of retinopathy between the 2 groups (*p*=0.05) with more advanced retinopathy observed in the VMIA group: Background retinopathy levels were higher in the No-VMIA group (41% vs 22%) and higher levels of proliferative disease in the VMIA group (16% vs 36%).

106 (89%) patients received aflibercept and 13 (11%) received ranibizumab as anti-VEGF treatment.

There was a mean of 7 injections after 12 months with no statistical difference between the groups (*p*=0.75)

At baseline 54/119 eyes (46%) had VMIA. There was resolution in 2 patients and development in 4, indicating a net gain of VMIA in 2 patients over 12 months. Prevalence of VMIA at 12 months was therefore 56/119 (47%).

**Fig. 1:**
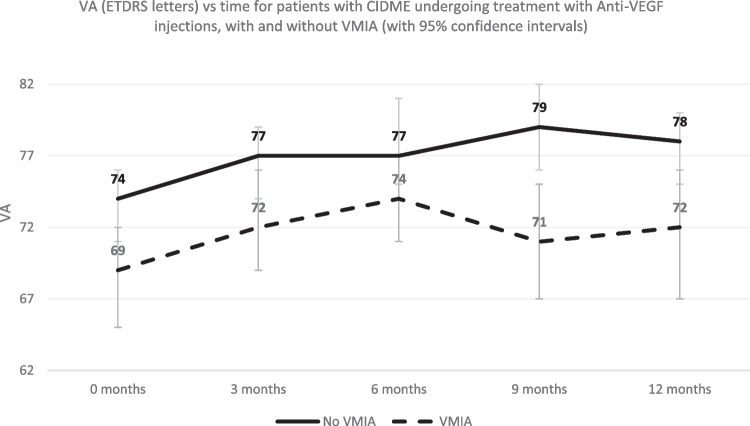
Line graph of visual acuity (VA) in ETDRS letters vs time (months)

**Fig. 2:**
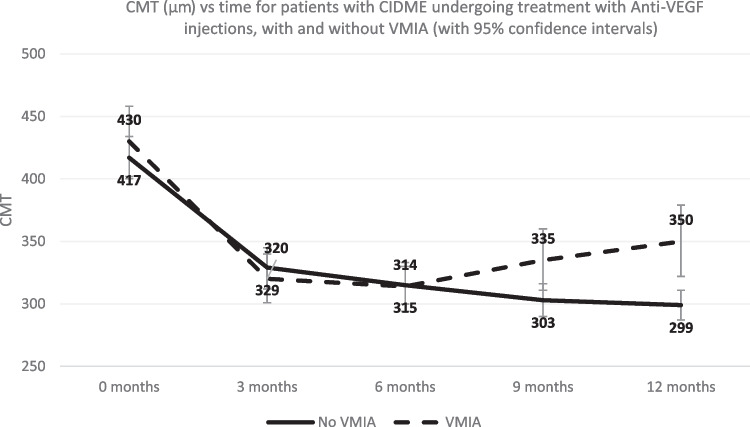
Line graph of central macular thickness (CMT) in microns change vs time (months)

The baseline acuity, CMT and the response to treatment are presented in Table 2. Chronological VA and CMT change are represented graphically in Figs. 1 and 2.

**Table 2:** Response to treatment at quarterly intervals. VA measurements in ETDRS letters and Mean CMT change is presented for the whole cohort and subdivided by presence or absence of VMIA along with Median CMT change from baseline (in microns)

	All subjects	No VMIA	VMIA	P-value
Mean VA Baseline (SD)	71 (12)	74 (11)	69 (12)	**0.03**
Mean VA 3/12 (SD)	75 (12)	77 (10)	72 (13)	**0.03**
Mean VA 6/12 (SD)	76 (11)	77 (11)	74 (12)	0.15
Mean VA 9/12 (SD)	75 (13)	79 (10)	71 (14)	**0.0007**
Mean VA 12/12 (SD)	75 (13)	78 (9)	72 (16)	**0.015**
Mean CMT baseline (SD)	423 (86)	417 (68)	430 (103)	0.43
Mean CMT 3/12 (SD)	325 (63)	329 (63)	320 (64)	0.51
Mean 6/12 (SD)	314 (64)	315 (59)	314 (71)	0.91
Mean 9/12 (SD)	318 (67)	303 (45)	335 (82)	0.19
Mean 12/12 (SD)	322 (84)	299 (49)	350 (105)	**0.0006**
Median Baseline CMT (IQR)	409 (371-466)	407 (368-455)	416 (372-467)	0.43
Median CMT change 3/12 (IQR)	84 (138 - 35)	77 (103-36)	94 (163-35)	0.45
Median CMT change 6/12 (IQR)	90 (136-52)	89 (131-55)	90 (172 -39)	0.86
Median CMT change 9/12 (IQR)	101 (148-47)	110 (147-69)	74 (163-26)	0.06
Median CMT change 12/12 (IQR)	101 (144-61)	108 (140-75)	79 (144-22)	**0.04**

*SD* standard deviation, *IQR* interquartile range

In summary, baseline VA was statistically better in the No-VMIA group. Both cohorts demonstrated visual improvement until 6 months. Hereafter VA continued to improve in the No-VMIA group while deteriorating in the VMIA group.

Baseline CMT measurements were comparable at baseline (417μ and 430μ in the No-VMIA and VMIA groups respectively) and improved to approximately 300μm in both groups. From 6 months CMT continued to improve in the no-VMIA while progressively deteriorating in the VMIA group. Change in CMT was statistically different at 12 months between the 2 groups (108μm and 79μm, *p*=0.04)

## Discussion

This study has provided evidence to support the previous suggestion of a differential response in VA and CMT outcomes in patients with and without VMIA, completing 1 year of anti-VEGF treatment for CIDME.

Initial response from baseline to 6 months was similar between patients with and without VMIA. However, between 6- and 12-months VA and CMT continued to improve in patients without VMIA while deterioration was observed in patients with interface abnormality.

The divergence in response, with possible worsening of acuity and CMT in the VMIA group and continued improvement in both parameters in the no-VMIA group from 6-12 months, has not previously been either investigated for or identified. This may be because this is the first study in which CMT and VA have been analysed in reasonably large numbers and at regular intervals across a 12 month follow-up period.

There are four other studies examining relationship between presence of VMIA and response to anti-VEGF treatment, In 2 of these studies patient numbers are relatively small (*n*=24 and 11) with short follow-up times of 1 and 3 months respectively [2, 3]. None of the published studies reported VA and CMT findings at more than one point in time.

Wong and colleagues published outcomes of 104 eyes from 77 patients analysing CMT and VA response to ranibizumab at baseline and 12 months [1]. There were many consistencies between their findings and the outcomes of this study: the sample size, injection number and demographics were comparable with similar rates of VMIA reported at baseline (43% vs 46% in this study). They also described worse acuity and anatomical outcomes in patients with VMIA at 12 months, with greater CMT reduction in patients without VMIA. However their outcomes measures are only described at 12 months and do not report the intermediate changes at quarterly intervals.

Mikhail et al reviewed 146 eyes of 100 patients treated with ranibizumab for CIDME. Contrastingly the authors reported no statistically significant association between baseline VMIA and outcome to ranibizumab treatment [10]. However, the mean follow-up time was only 9 months. Yoon et al examined the response of 15 eyes of 11 patients, with and without VMIA, between baseline and 3 months [3]. Although the sample size was small, marked CMT reduction was demonstrated in both groups.

The variation in follow up times across these studies may explain the difference in outcomes.

In this study, the early CMT response was comparable between both groups at baseline, 3 months, and nearly identical at 6 months. CMT subsequently increased in the VMIA group while continuing to improve in the No-VMIA group over the latter six months. By 12 months the VMIA group were 51 microns thicker compared to the No-VMIA group. These responses may offer fresh insight into the pattern of response to anti-VEGF treatment in eyes with and without VMIA.

The role of the vitreous and vitreomacular interface on the development and response to treatment in DMO has been speculated to arise from micro and macro traction, a reservoir for pro-inflammatory factors ‘trapped’ in the retina and as a barrier to therapeutic anti-VEGF drug diffusion [9, 11, 12] VMIA has also been suggested to be a marker of more severe baseline activity of diabetic retinopathy and to occur in eyes with more limited visual potential [1].

This study recognised a statistically higher proportion of advanced diabetic retinopathy in patients with VMIA compared to the no-VMIA group. The sustained significant difference in VA from baseline between the groups, may support the association between disease severity and limited visual potential in presence of VMIA.

The theory VMIA acts as a barrier to anti-VEGF drug diffusion is challenged by the early CMT response to treatment in patients with VMIA. It would therefore seem reasonable to conclude that the observed differences were not due to failure of anti-VEGF drugs to work in eyes with VMIA but rather a difference in behaviour in these eyes between 6 and 12 months.

Undertreatment is a consideration: 90% of patients were treated with fixed-dosed aflibercept as opposed the treat and extend regime for ranibizumab treated patients. It is therefore possible some patients on fixed dosing regimens would have met treat and extend treatment criteria. However as these patients were not assessed from a treat and extend perspective in the first instance it is not possible to retrospectively apply these treatment criteria.

Fixed dosing was, however, applied equally and without prejudice to both groups irrespective of baseline VMIA status and, as anticipated with fixed dosing, mean injection was the same in both groups.

We therefore believe that systematic under treatment is an unlikely explanation for the observed finding. It may however be that better results would have been obtained with greater treatment in the VMIA group and warrants further focused research.

Another potential explanation for the worsening of parameters observed in eyes in interface abnormality is the cellular effect of VMIA in the presence of anti-VEGF agents.

The OCT appearance forms the basis for diagnosis and classification of VMIA, [9] however Hagenau et al have reported the presence of histological interface abnormalities, prior to a tractional lesion being evident on OCT scanning [13]. They investigated the histological appearance of ILM peeled from 27 eyes undergoing PPV for DME and reported that all analysed eyes demonstrated evidence of VMIA irrespective of OCT classification prior to surgery. Eyes with visible VMIA on OCT were found to have greater and more complex cellular membranes, suggesting VMIA is likely to represent a spectrum of disease.

At one end of this spectrum is the angio-fibrotic switch, which is described as a shift in the balance of VEGF and connective tissue growth factor (CTGF) that leads to increasing fibrosis [14, 15]. Use of anti-VEGF agents potentiates this imbalance and pro-fibrotic CTGF may promote retinal contracture. In instances where initial VEGF levels are lower it may be that the response is subtler.

Walsh et al reported the histological presence of contractile myofibroblasts in retinal membranes from patients with proliferative diabetic retinopathy (PDR). These cells are capable of retinal traction and ability to produce collagen [16]. Hagenau et al also identified the capacity of hyalocytes to transdifferentiate into myofibroblasts [13] and other authors have discussed progressive histological interface changes following anti-VEGF injections, with increasing traction and fibrosis [17, 18].

We did not analyse VMIA progression in our study, however this may be observed by Wong et al who report VMIA progression, from baseline to 12 months, in 25% of patients [1].

Changing cellular structures in the presence of anti-VEGF may, therefore, at least in part explain the poorer outcome in patients with VMIA after 6 months observed in this study.

There were limitations to our study. This was a retrospective, non-controlled, non-randomised study. Patients were not clinically examined specifically for this study so rates of posterior vitreous detachment (PVD), thought to be therapeutically beneficial in DME [19], have not been not recorded.

The ERM is this cohort is not graded and we have not quantified or measured retinal contracture, which would be expected if retinal traction were responsible. ERM grading is typically graded according to the disruption of the inner layers on OCT [20], however this proves difficult in the context of dual pathology where DMO also causes inner layer disruption.

VA are not best corrected and there was no specified protocol for recording acuity. Patients with documented cataract prior to treatment, or who underwent cataract surgery during the follow up period, were excluded from this study. We did not control for cataract progression, although we have no reason to believe rates of progression would be preferentially higher in either group. Systemic factors including HbA1c, which have been shown in some studies to be associated with poorer response to anti-VEGF, were also not recorded [21] . Our follow up period was limited to 12 months.

Despite these limitations there are significant and important observations taken from the real-world clinical setting and, to the best of our knowledge, this study comprises the largest cohort of patients examined for this condition.

In conclusion, our study has shown a 46% incidence of VMIA amongst patients newly diagnosed with centre involving DME undergoing treatment with anti-VEGF injections. The prevalence increased to 47% by 12 months. We have also demonstrated a significant difference in CMT and VA response to anti-VEGF treatment in patients with and without VMIA. Initial response was similar between the 2 groups up until 6 months. From 6 to 12 months significant differences in treatment response emerged.

Differences in clinical response between patients with and without VMIA may help guide further prospective controlled studies and optimise treatment strategies.

## Supplementary information

ESM 1 (DOCX 827 kb)
