# Supplementary material for: The impact of Vitreo-Macular interface abnormalities on the response to Anti-VEGF therapy for centre involving diabetic macular oedema
Source: Graefes Arch Clin Exp Ophthalmol. 2024 May 21;262(11):3501–8. doi: 10.1007/s00417-024-06518-6 (PMC11584421; doi:10.1007/s00417-024-06518-6)

Image 1

OCT appearance of a) DMO without VMIA, b) DMO with ERM and c) DMO with VMT ERM. ERM was defined as presence of a hyperreflective band at the ILM layer with underlying corrugation of the inner retinal layers. VMT was defined as cortical vitreous detachment with foveal attachment and associated foveal anatomical distortion


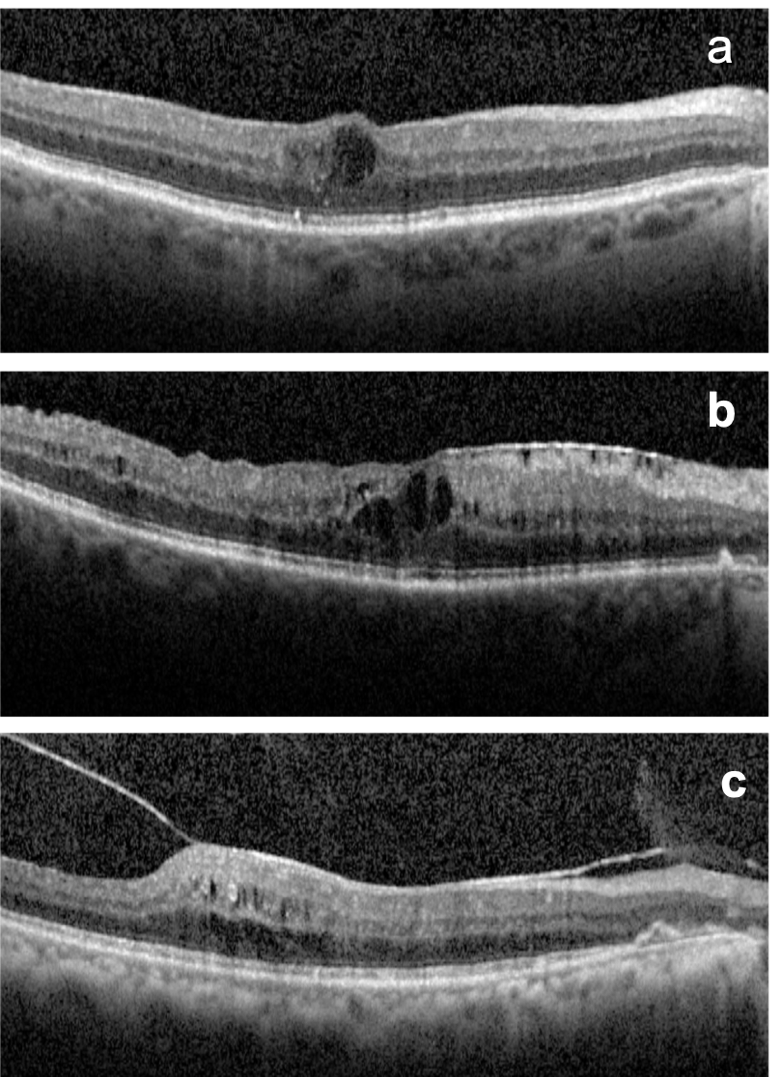

Supplement: Supplementary file 1 — (DOCX 827 kb) [file 417_2024_6518_MOESM1_ESM.docx]
